# Complement factor D is linked to platelet activation in human and rodent sepsis

**DOI:** 10.1186/s40635-021-00405-8

**Published:** 2021-08-16

**Authors:** O. Sommerfeld, K. Dahlke, M. Sossdorf, R. A. Claus, A. Scherag, M. Bauer, F. Bloos

**Affiliations:** 1 Department of Anesthesiology and Intensive Care Medicine, Jena University Hospital, Jena, Germany; 2 Center for Sepsis Control and Care (CSCC), Jena University Hospital, Jena, Germany; 3 Institute of Medical Statistics, Computer and Data Sciences, Jena University Hospital, Jena, Germany

**Keywords:** Alternative complement pathway, Complement factor D, Coagulopathy, Infection

## Abstract

**Background:**

The complement factor D (CFD) exerts a regulatory role during infection. However, its physiological function in coagulopathy and its impact on the course of an infection remains unclear.

**Materials:**

Wild-type and CFD-deficient mice (*n* = 91) were subjected to cecal ligation and puncture to induce sepsis. At several time points, markers of coagulation and the host-immune response were determined. Furthermore, in patients (*n* = 79) with sepsis or SIRS, CFD levels were related to clinical characteristics, use of antiplatelet drugs and outcome.

**Results:**

Septic CFD-deficient mice displayed higher TAT complexes (*p* = 0.02), impaired maximal clot firmness, but no relevant platelet drop and reduced GPIIb/IIIa surface expression on platelets (*p* = 0.03) compared to septic wild-type mice. In humans, higher CFD levels (non-survivors, 5.0 µg/ml to survivors, 3.6 µg/ml; *p* = 0.015) were associated with organ failure (SOFA score: *r* = 0.33; *p* = 0.003) and mortality (75% percentile, 61.1% to 25% percentile, 26.3%). CFD level was lower in patients with antiplatelet drugs (4.5–5.3 µg/ml) than in patients without.

**Conclusion:**

In mice, CFD is linked to pronounced platelet activation, depicted by higher GPIIb/IIIa surface expression in wild-type mice. This might be of clinical importance since high CFD plasma concentrations were also associated with increased mortality in sepsis patients.

**Supplementary Information:**

The online version contains supplementary material available at 10.1186/s40635-021-00405-8.

## Introduction

During infection, invading microorganisms activate the host response where the complement system is part of the “first line of defense”. Complement activation occurs via the classical, alternative or lectin pathways. A deficiency in one of the three pathways is associated with increased mortality, impaired bacterial clearance and high cytokine levels [1]. Several of such conditions are known: (1) genetic deficiencies of the classical pathway are associated with autoimmune diseases. Such diseases are accompanied with impaired humoral response as well as ineffective elimination of immune complexes, apoptotic materials, and necrotic debris [2]. (2) Deficiencies of components of the alternative (complement factor D, properdin) and of the lectin (MBL) pathways impair pathogen removal and increase the susceptibility to infections [2]. A high susceptibility to bacterial infections with *Neisseria spp.* has been observed in complement factor D (CFD) deficient humans [3, 4].

During systemic infections, the complement and coagulation system are tightly networking proteolytic cascades [5]. (1) Platelets and platelet-microparticles (PMP) are able to activate classical and terminal complement activation pathways [6, 7]. (2) Thrombin cleaves C5 through terminal complement activation [8]. (3) FXa, FXIa, and plasmin function as C3 and C5 convertase [9]. In addition, platelet adhesion to injured endothelial cells is promoted by the activated coagulation and complement system [10], i.e., the anaphylatoxin C5a induces tissue factor (TF) activity in human endothelial cells [11]. In turn, activated platelets mediate complement activation via P-selectin (CD62P) expression [12, 13]. Such interactions may also contribute to thrombosis and thrombocytopenia [14].

One serine protease of the alternative complement activation pathway is the complement factor D (CFD). CFD is stored in human platelets and is released after stimulation with ADP, collagen or thrombin [15]. The blockade of CFD with an anti-factor D antibody inhibits platelet activation by reduced CD62P expression on the platelet surface [16]. Otherwise, the physiological function of CFD in platelets and its role in coagulation during infection remains unclear. Based on the literature above, we designed a translational study in both mice and men to address the impact of CFD on coagulation in sepsis. We hypothesize (1) that CFD impacts coagulopathy in mice with systemic infection; (2) that CFD interaction with coagulation is associated with outcome in sepsis patients, and (3) that antiplatelet drugs affect the CFD level in patients with sepsis.

## Materials and methods

### Study design and interventions of mice experiments

All animal studies were reviewed and approved by the licensing committee of the state of Lower Saxony (Landesamt für Verbraucherschutz und Lebensmittelsicherheit, Oldenburg, Germany, NRW 550-8594-21-006-089-02), and were in accordance with the German and European guidelines on protection of animals. Our study was designed before the MQTiPSS guidelines for improving animal modeling in sepsis were published [17]. However, we were complying with most of the recommendations regarding study design, human modeling, infection types and organ failure/dysfunction.

Male complement factor D knockout mice (fD^−/−^) were generated as described by Xu et al. [18], on the background of C57BL/6J. For control studies, male C57BL/6J mice (wild-type) were used. Animals were bred and housed at the animal facility of the Jena University Hospital. For the studies mice were 22 g (± 3 g) and 12–14 weeks old. Throughout all experiments, animals were kept under standardized conditions with access to food and water ad libitum.

Cecal ligation and puncture (CLP) was performed as described in detail previously [19]. Mice were anesthetized by i.p. injection of ketamine (Ketamin, DeltaSelect, Dreieich, Germany) and xylazine (Rompun, Bayer, Leverkusen, Germany) at a dose of 1 µl/g body weight (BW) (mixture of 10 (ketamine) to 1 (xylazine)). To shortly sum the procedure, after disinfection of the abdominal area the mice were laparotomized by midline incision. Then, 2/3 of the exposed cecum was ligated and punctured through and through with a 21-gauge needle [1]. Finally, wound closure was performed by applying simple running sutures to the abdominal musculature and the skin. For the current study, CFD-deficient mice and wild-type mice as comparable control group were undergoing CLP procedure and scored every 3 h, applying the clinical severity score described before [20]. At indicated time points (control, 3, 6 and 24 h = 4 groups/genotype) the animals were euthanized and whole blood was drawn. Therefore, anesthetized sham control mice or septic mice were laparotomized and blood was taken by heart puncture.

Blood cell count and sample preparation in mice experiments

Whole blood was taken from septic (six and 24 h after CLP) and control mice of both genotypes (fD^−/−^, wild-type) for full-automatic analysis of leukocytes and platelets counts (PocH-100iV Diff; Sysmex, Leipzig, Germany). The samples for the ROTEM and FACS analyses were used directly after blood drawing. Platelet-rich-plasma (PRP) was prepared by centrifugation at 180 g for 12 min at room temperature. Afterwards the PRP was obtain carefully for further analyses. During sample preparation the PRP were kept on 4 °C to avoid platelets activation. For plasma preparation whole blood was centrifuged at 2000×*g* for 10 min. Afterwards, samples were snap-frozen and stored at − 80 °C for further analysis. For the ELISA tests, plasma samples were thawed on ice before sample preparation.

### Rotational thromboelastometry (ROTEM^®^, TEM) in mice experiments

For thromboelastometry (TEM) citrate anticoagulated whole blood from control and septic animals of both genotypes was used (blood volume adapted mixture (10:1) of 9 part of whole blood and 1 part of 3.2% tri-natrium-citrate solution). Rapidly after sampling whole blood EXTEM (extrinsic coagulation pathway), INTEM (intrinsic coagulation pathway) and FIBTEM (fibrinogen function) test analyses of each group were performed with the ROTEM®delta system (Tem Innovations GmbH, ROTEM^®^delta system [000200100-DE], EXTEM assay reagents [503-13-20], INTEM assay reagents [503-12-20] and FIBTEM assay reagents [503-16-20] Munich, Germany) according to the manufacturer’s instructions.

### ELISA of D-dimer and TAT complexes in mice experiments

D-dimer concentration (Mouse D-dimer ELISA kit [CEA506Mu], Uscn Life Science Inc., Wuhan, China), TAT complexes (Mouse Thrombin–Antithrombin complex ELISA kit [SEA831Mu], Uscn Life Science Inc., Wuhan, China) were measured in septic and control mice plasma of each genotype (wild-type and fD^−/−^). The minimum of detectable dose of mouse D-dimer was 0.54 ng/ml and for mouse TAT complexes was 0.004 ng/ml. The ELISAs were performed according to the manufacturer’s instructions.

### Flow cytometry of GPIIb/IIIa and P-selectin surface expression and cytokine levels (CBA assay) in mice experiments

GPIIb/IIIa and P-selectin surface expression were detected by flow cytometry. Therefore platelet-rich plasma (PRP) was prepared by centrifugation (see above) and stained with antibody for activated form of GPIIb/IIIa (PE Integrin alphaIIbbeta3 [M025-2], 5 µl antibody solution to stain ~ 10^6^ platelets in a recommended volume of 25 µl, recalcification using Tyrode–Hepes buffer containing 1 mM CaCl2 before flow cytometric analysis, Emfret Analytics, Eibelstadt, Germany) and P-selectin (FITC Rat Anti-mouse CD62P [553744], concentration 0.5 mg/ml, Becton Dickinson (BD), East Rutherford, USA) followed by flow cytometric analysis. Plasma cytokine levels were measured using CBA assay (Cytometric Bead Array (CBA) Mouse Inflammation [552364], Becton Dickinson (BD), East Rutherford, USA) by FACS. The plasma samples were collected and analyzed according to the manufacturer’s instructions.

### Study design and human study population

The study was conducted in accordance with Helsinki Declaration and approved by the local ethical committee of Jena University Hospital (474403/16). Plasma samples were collected from ICU patients between 2001 and 2005.

Plasma samples were stored at − 80 °C in the Integrated BioBank Jena (Jena University Hospital, Germany). Informed consent was obtained from all patients or their legal representatives. Inclusion criteria for this analysis were either new onset of sepsis (for the trial patients were evaluated based on the definition of sepsis-1) [21] or presence of systemic inflammation after cardiopulmonary bypass for cardiac surgery. Plasma samples had to be available between 24 and 48 h after diagnosis of sepsis or SIRS. Patients, who had received fresh frozen plasma or thrombocyte transfusion during their ICU stay, were excluded from this analysis. In total *N* = 79 patients were included in the study, whereas the study cohort was divided into two groups: group (1) N = 45 infected patients (= diagnosis: sepsis) and group; (2) N = 35 non-infected patients (= diagnosis: SIRS). Several parameters were provided from the study group (demographical data, fibrinogen level, C-reactive protein, SOFA [Sepsis-related Organ Failure Assessment] score [22]). The medication history of the study population was evaluated with respect of the use of antiplatelet drugs, especially acetylsalicylic acid (ASA) or clopidogrel. Further, we defined cut-off values of measured complement factor D plasma levels: < 25% percentiles (CFD levels ≤ 2.4 µg/ml), > 25% and < 75% percentiles (CFD levels > 2.4 µg/ml and < 5.0 µg/ml), > 75% percentiles (CFD level ≥ 5.0 µg/ml).

### ELISA of D-dimer, TAT complexes and complement factor D (CFD) in the human study population

D-dimer (D-Dimer Human Simple Step ELISA^®^ Kit [ab260076], abcam^®^, Cambridge UK), TAT complexes (Human Thrombin–Antithrombin Complex ELISA Kit (TAT) [ab108907], abcam®, Cambridge UK) and complement factor D (Quantikine^®^ Human Complement Factor D Immunoassay [DFD00], R&D Systems^®^, Minneapolis USA) levels were measured in human plasma. The minimum of detectable dose of human D-dimer was 0.07 ng/ml, for human TAT complexes was 1.5 ng/ml, and for human CFD was 0.013 ng/ml. Therefore, ELISAs were performed according to the manufacturer’s instructions.

### Statistical analyses

For the animal study, after descriptive statistical analyses, non-parametric tests using Wilcoxon–Mann–Whitney test, or the Kruskal–Wallis test (Dunn’s test correction) were performed. For the human study, after descriptive statistical analyses, the obtain data were assessed for normal distribution. For data following normal distribution, we applied parametric tests (Student’s *t*-test). Otherwise, non-parametric tests (Wilcoxon–Mann–Whitney test or the Kruskal–Wallis test with Dunn’s test correction) were used. We report bivariate Pearson correlation coefficients for several variables (CFD, TAT complexes, D-dimer, platelets, SOFA score, modified DIC score). We investigated the association of CFD levels measured after ICU admission and diagnosis sepsis or SIRS for time to in-hospital mortality. The estimated percentiles (25, 50 and 75 percentiles) of the calculated mean CFD plasma level (with confidence interval, CI) of all patients were defined as cut-off limits. Three groups were conducted and the CFD value of each patient of both groups (SIRS and Sepsis) assigned to the groups. Results were displayed in a Kaplan–Meier curve with the classification for CFD levels based on the calculated percentiles (25%, 50% and 75%). Using univariate and multivariable Cox regression models, we extended the investigation of CFD level (linear) with time to in-hospital mortality including demographic (sex, age) and clinical variables (TAT complexes, D-dimer, platelets) as well as ICU scores (SOFA, modified DIC score) and state of infection (subgroup sepsis). In multivariate analysis, we adjusted for age and SOFA score which are known confounders when assessing effects on hospital mortality. From these models, we derive hazard ratios (HR) and confidence intervals (CI) with a coverage of 95%. We applied an explorative two-sided significance level of α = 0.05 and did not correct for multiple testing. Results are depicted as bar plots (mean + SEM) or boxplots (interquartile range [IQR], 0.25, 0.75; whiskers: min, max). Individual data points (*n*) are plotted over the boxes, or the n was specified otherwise in the bars or figure legend. The results for patient characteristics are stated as mean with range or min to max. All analyses were done using GraphPad Prism 6 or IBM SPSS 24.

## Results

### Coagulation and platelets function in septic fD^−/−^ and wild-type mice

**Fig. 1:**
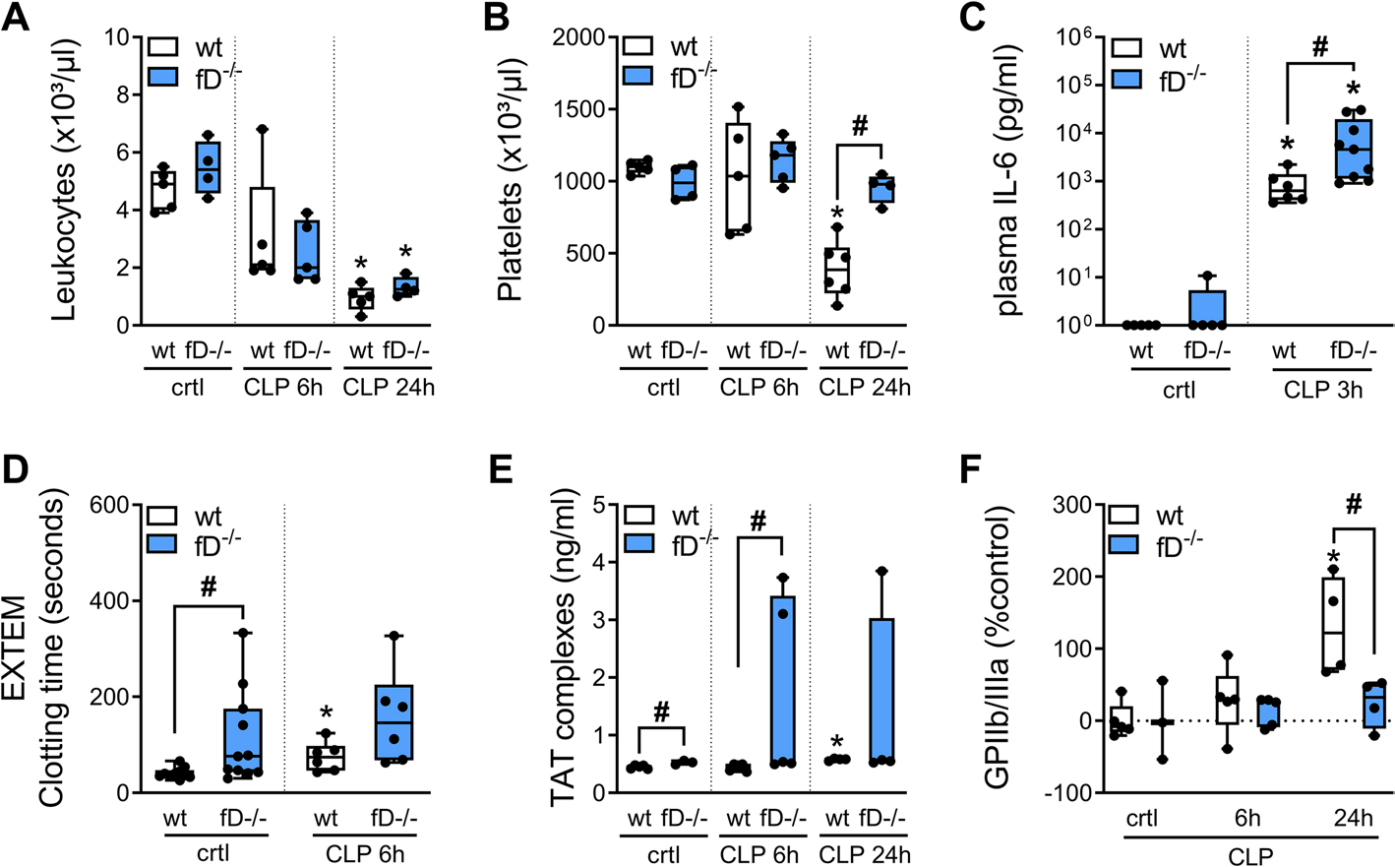
Complement factor affects immune response and platelets function. **A**, **B** Whole blood leukocytes dropped in septic mice (both groups) after CLP. No platelet drop was found in septic fD^−/−^ mice (fD^−/−^) 24 h after CLP, whereas platelet counts were reduced in septic wild-type (wt) at same time point. **C** Plasma cytokine levels of IL-6 were significant higher in septic fD^−/−^ mice compared to wild-type three hours after CLP. **D** EXTEM test were performed and clotting time (ct) in fD^−/−^ mice (fD-/-) was prolonged in both sham control (crtl) and after CLP. **E** TAT complexes were found to be higher in control (crtl) and septic fD^−/−^ mice. **F** Platelet surface expression of GPIIb/IIIa was reduced in septic fD^−/−^ mice compared to wild-type 24 h after CLP. Statistics: *significant to fD^−/−^ sham control (crtl) or wild-type (wt) sham control (crtl) with *p* < 0.05, (**A**, **B**, **E** and **F**) Kruskal–Wallis test (Dunn’s test correction) or (**C** and **D**) Wilcoxon–Mann–Whitney test. #Statistical significance (*p* < 0.05) between fD^−/−^ and wild-type (wt) by means of (**A** to **F**) Wilcoxon–Mann–Whitney test. Three to 12 mice/group

Leukocyte depletion was found in CFD-deficient (fD^−/−^) and wild-type (wt) mice 24-h after CLP (wt: *p* = 0.0003; fD^−/−^: *p* = 0.0004) (Fig. 1A). Cytokine levels increased in both groups after sepsis onset (wt: IL-6, *p* = 0.004; TNF-α, *p* = 0.008; IL-10, *p* = 0.008; fD^−/−^: IL-6, *p* = 0.001; TNF- α, *p* = 0.002; IL-10, *p* = 0.001), whereas IL-6 levels were higher in fD^−/−^ mice compared to wild-type at 3-h (*p* = 0.012) after CLP (Fig. 1B and Additional file 1: Figure S1D). Platelets counts dropped in wild-type mice (*p* = 0.003) but not in fD^−/−^ mice 24-h after sepsis induction (Fig. 1A). D-dimer concentrations increased continuously in both groups reaching highest values 24-h after sepsis onset (wt: *p* < 0.0001; fD^−/−^: *p* = 0.0006; Additional file 1: Figure S1C). We observed higher values of TAT complexes in control and septic fD^−/−^ mice (*p* = 0.02; Fig. 1D).

All three test settings of rotational thromboelastometry, i.e., EXTEM, INTEM and FIBTEM resulted in enhanced clotting times (ct) in sham control and septic fD^−/−^ mice. Only the EXTEM ct of sham control fD^−/−^ mice, which assesses the extrinsic pathway, differed from the wild-type sham control (Fig. 1C, Additional file 1: Figure S1A). CLP was associated with a reduction in maximal clot firmness (mcf) in all three test settings (EXTEM, INTEM and FIBTEM test) in both groups (wt: EXTEM, *p* = 0.002; INTEM, *p* = 0.03; FIBTEM, *p* = 0.002; fD^−/−^: EXTEM, *p* = 0.03; INTEM, *p* = 0.004; FIBTEM, *p* = 0.009). Further, INTEM and FIBTEM test revealed a lower mcf in septic fD^−/−^ mice compared to septic wild-type mice (for FIBTEM, Additional file 1: Figure S1B). P-selectin expression on isolated platelets increased in both groups after CLP over time but was more pronounced in wild type 24 h after sepsis induction compared to fD^−/−^ mice at that time point (wt: *p* = 0.008; fD^−/−^: *p* = 0.02; Additional file 1: Figure S1E). GPIIb/IIIa expression on isolated platelets of septic fD^−/−^ mice did not change relevantly during the experiment, while receptor expression of activated GPIIb/IIIa was enhanced on platelets of wild-type mice 24-h after CLP (*p* = 0.03; Fig. 1E) compared to septic fD^−/−^ mice at that time point and the wild-type control group.

### Clinical characteristics in the human study population

Patient characteristics are summarized in Tables 1, and 2. In short, the sample included a similar number of males and females with a mean age of 66 years (range 29–92 years). Their overall in-hospital mortality was 41%. The most frequent foci were pulmonal (22%) and abdominal (17%) infections.

**Table 1:** Patient characteristics and pathologies in the investigated study population

Characteristics	*N*			Infection	*N*
	Overall	Sepsis	SIRS		
Sex				Sepsis (%)	44 (56)
Female	40	22	18	Endocarditis	4 (5)
Male	39	22	17	Pneumonia	17 (22)
Age y				Intra-abdominal	13 (16)
Mean	66	66	65	Pneumonia/intra-abdominal	4 (5)
Range	29–92	31–92	29–88	Other	6 (8)
Outcome				SIRS (%)	35 (44)
Dead	32	23	9		
Survived	47	21	26	Total	79 (100)

**Table 2:** Patient characteristics and scores after diagnosis of sepsis or SIRS in the investigated study population

Variables	Overall		Sepsis		SIRS	
	*N*	Mean (min to max)	*N*	Mean (min to max)	*N*	Mean (min to max)
In-hospital stay (days)	78	27.2 (3–127)	43	34.0 (6–127)	35	18.7 (3–70)
CFD (µg/ml)	79	4.2 (0.2–15.0)	44	4.5 (0.8–15.0)	35	3.8 (0.2–11.2)
TAT complexes (ng/ml)	79	18.3 (4.7–56.8)	44	20.1 (5.6–56.8)	35	16.0 (4.7–43.7)
D-dimer (µg/ml)	79	3.4 (0.4–29.8)	44	4.8 (0.6–29.8)	35	1.7 (0.3–6.3)
Platelets (Gpt/l)	79	190.7 (25–851)	44	199.7 (25–851)	35	179.3 (69–515)
Fibrinogen (g/l)	73	4.5 (1.3–10.0)	39	5.4 (2.1–10)	34	3.4 (1.3–8.6)
CRP (mg/l)	76	133.7 (4.9–500.0)	44	191.1 (15–500)	32	54.8 (4.9–229)
SOFA score	78	8 (1–15)	44	9 (5–15)	35	7 (1–14)
DIC score (modified)	79	3 (2–7)	44	3 (2–7)	35	3 (2–4)

### Complement factor D (CFD) levels and their association with antiplatelet drugs in the human study population

The mean level of complement factor D (CFD) was 4.2 µg/ml (range 0.2–15.0 µg/ml, Table 2) at ICU admission. In septic patients the mean CFD value (4.5 µg/ml, Table 2) was higher compared to SIRS patients (3.8 µg/ml, Table 2).

**Fig. 2:**
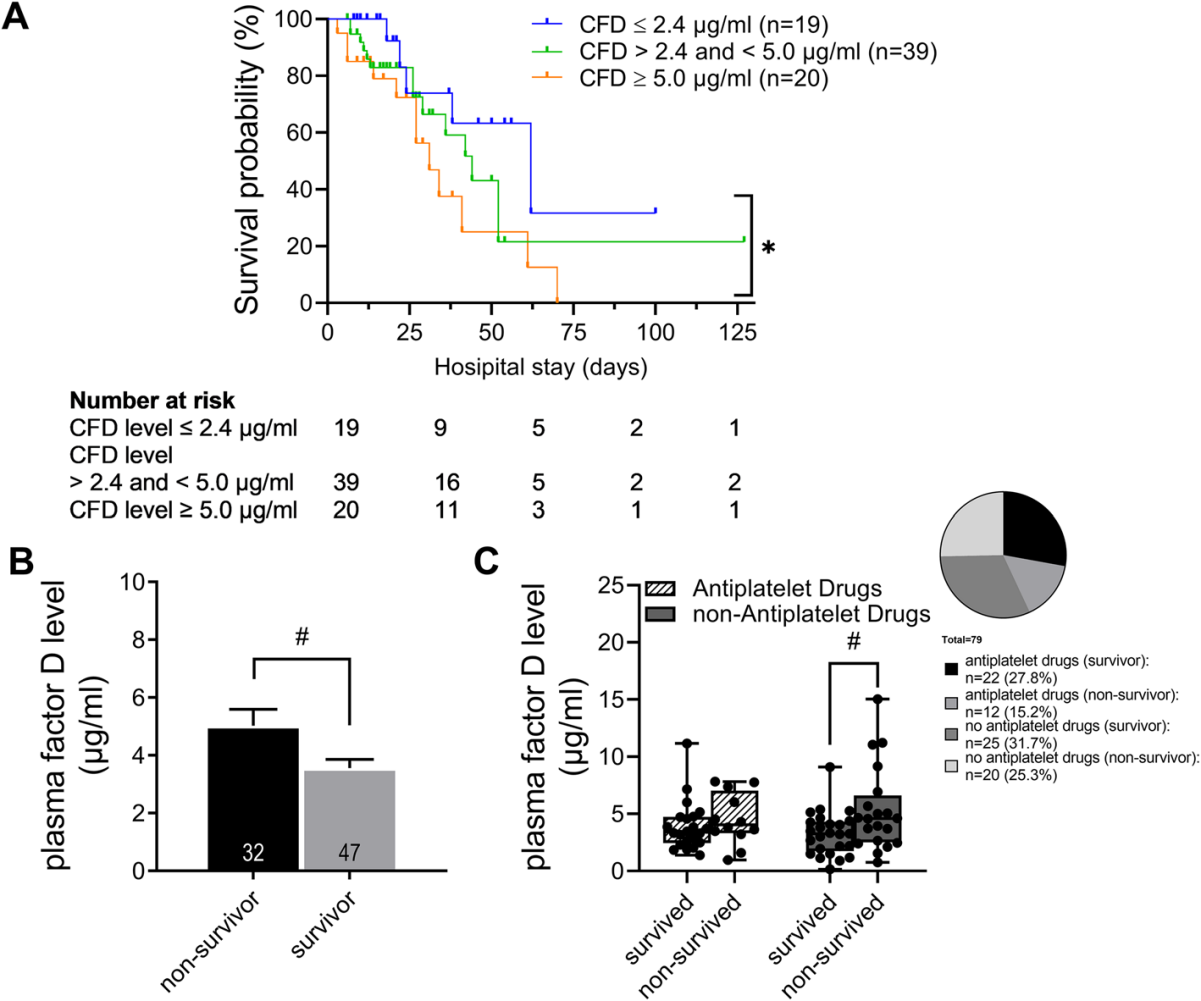
Height of complement factor D level predicts disease severity. **A** Kaplan–Meier curve: cut-off values were estimated from 25th, 50th and 75th percentile. Complement factor D (CFD) levels above 75th percentile were associated with earlier and higher in-hospital mortality. **B** Complement factor D: highest mortality was associated with significant higher complement factor D level in non-survivors compared to survivor group. **C** Non-survivors with no antiplatelet drugs showed significantly higher CFD plasma level compared to patient who survived. Statistics: *statistical difference between indicated groups with *p* < 0.05. **A** Log-rank test. #Statistical significance (*p* < 0.05) between non-survivor and survivors by means of **B **Student’s *t*-test or **C** Wilcoxon–Mann–Whitney. In total 79 patients

The plasma CFD concentration was increased in non-survivors compared to survivors (5.0 µg/ml in non-survivors to 3.6 µg/ml in survivors, *p* = 0.015; Fig. 2B) at ICU admission. The plasma CFD value of non-survivors and survivors were also compared in both subgroups. Septic non-survivors displayed on average 1.8 µg/ml higher CFD value compared to septic survivors (*p* = 0.042; Additional file 1: Figure S2A). In SIRS patients, CFD value on average differed between survivors and non-survivors by 0.7 µg/ml. The CFD concentration of non-survivors without antiplatelet drugs were higher compared to non-survivors with antiplatelet drugs (acetylsalicylic acid [ASA] or clopidogrel; 5.3 µg/ml in patients with no antiplatelet drugs to 4.5 µg/ml in patients with antiplatelet drugs). Non-survivor without antiplatelet drugs displayed significantly higher CFD concentration compared to the survivor with no antiplatelet drugs (5.3 µg/ml in non-survivors to 3.3 µg/ml in survivors, *p* = 0.044; Fig. 2C).

### Complement factor D (CFD) compared to coagulation parameters and scores of organ dysfunction in the human study population

D-dimer level and SOFA score were higher in non-survivor compared to survivors in SIRS patients without reaching statistical significance. In sepsis patients, D-dimer level and SOFA score were elevated without being different between non-survivors and survivors (Additional file 1: Figure S2B, S2C). For TAT complexes and modified DIC score, we observed no evidence for differences between the subgroups (Additional file 1: Figure S2B, S2C). During the first 5 days after ICU admission, platelet counts showed minor differences depending on low (≤ 2.4 µg/ml), mid (> 2.4 µg/ml and < 5.0 µg/ml) or high (≥ 5.0 µg/ml) CFD plasma concentration (Additional file 1: Figure S2D). Factor D concentration were positively correlated with the SOFA-score (*r* = 0.33, *p* = 0.003). Platelet counts concentrations were positively correlated with TAT complexes (*r* = 0.32, *p* = 0.004) and negatively correlated with both SOFA-score and modified DIC score (*r* = − 0.23, *p* = 0.04; *r* = − 0.23, *p* = 0.04; Additional file 1: Table S1, Figure S3).

### Complement factor D (CFD) to predict in-hospital mortality in the human study population

We found higher CFD levels to be associated with an increased in-hospital mortality (*p* = 0.03, Table 3 and Fig. 2A).

**Table 3:** Association of complement factor D levels to in-hospital mortality

CFD level	*N* (non-survivor)	Percentile	Median survival	95% CI
Group 1: ≤ 2.4 µg/ml	19 (5)	25th	62	(26.949–97.051)
Group 2: > 2.4 and < 5.0 µg/ml	39 (14)	50th	44	(31.494–56.506)
Group 3: ≥ 5.0 µg/ml	20 (12)	75th	31	(20.994–41.006)
Total			42	(32.291–51.709)

In the Cox regression analysis, depicted in Table 4, complement factor D (CFD) concentration was independently associated with overall survival when adjusting for age and SOFA score (Table 4).

**Table 4:** Regression model results for in-hospital mortality in 78 patients with diagnosis sepsis or SIRS

Variables	Cox regressions model for in-hospital mortality					
	Univariate models			Multivariable models		
	Hazard ratio	95% CI	*p* value	Hazard ratio	95% CI	*p* value
CFD	1.13	(1.03–1.25)	0.013	1.13	(1.00–1.28)	0.043
Age	1.03	(1.00–1.06)	0.049	1.02	(0.99–1.06)	0.092
Sex	1.62	(0.78–3.36)	0.193			
TAT complexes	0.99	(0.96–1.03)	0.679			
D-dimer	1.02	(0.93–1.13)	0.636			
Platelets	1.00	(0.99–1.00)	0.512			
SOFA score	1.05	(0.91–1.21)	0.548	0.99	(0.84–1.16)	0.869
DIC score (modified)	1.22	(0.80–1.87)	0.360			
Sepsis	0.89	(0.40–2.00)	0.784			

Statistics: univariate and multivariable Cox regression models (linear) and derived hazard ratios (HR) and confidence intervals (CI) with a coverage of 95% of demographic and clinical variables (*p* < 0.05)

## Discussion

This translational study supports an important role for the serine protease complement factor D (CFD) in sepsis, both in the mouse as well as in humans: in mice, CFD is associated with a significant impact on coagulation by enhanced platelet function in septic wild-type mice. In humans, high CFD plasma concentration was associated with an increased mortality in sepsis and antiplatelet drugs might favorably outcome affect the CFD plasma level.

While the crosslink between coagulation and complement system is well established [3–5, 7, 10–13, 23], the impact of CFD on the coagulation cascade is still unclear. It is known that CFD is localized in human platelets [15]. Our data add important insights regarding the functional role: an enhanced thrombin generation reflected by higher TAT complex concentration in CFD-deficient mice is not only linked to compensatory C5a formation during sepsis, but also contributes to abnormal coagulation activity. Thrombin is known as a potent platelet activator and initiates GPIIb/IIIa expression on thrombocytes [24]. Interestingly, the surface receptor expression of GPIIb/IIIa was significantly reduced in CFD-deficient mice, while we observed an increase in thrombocyte surface markers in wild-type mice. Other markers of coagulation such as the results of ROTEM (clotting time, maximal clot firmness) or D-dimer were affected in CFD-deficient as well as in wild-type mice. So, during infection CFD significantly impacts the inflammation-triggered activation of the coagulation cascade in mice, which was found in wild-type mice by pronounced surface expression of GPIIb/IIIa, P-selectin and the decrease of platelets and hint to the hypercoagulable state during sepsis.

The classical and alternative complement pathways play an essential role to clear endotoxin and to act as critical defense against bacterial infection [25]. Especially in humans, deficiency in factors of the alternative complement pathway such as CFD or properdin is reported to be associated with meningococcal infection and fulminant septic shock [3, 4, 26, 27]. In mice, CFD deficiency is associated with the loss of the regulatory role of the alternative pathway. Factor D-deficient mice showed reduced bacterial clearance, increased cytokine levels and fatal outcome [1].

In humans, we found high CFD levels in non-survivors of the whole study population and selectively in sepsis patients. These high CFD levels represent a strong activation of the alternative pathway. Alternative complement activation acts as an amplification loop triggering the hyperactive inflammatory response and thereby contributing negatively to the progression of the disease [18, 28–30]. Furthermore, CFD is a modulator of complement activation during infection affecting the innate immune response [1]. This might be the reason why a high CFD generation as observed in our study was associated with a high in-hospital mortality. Further, the positive correlation between SOFA score and CFD would suggest a negative effect regarding (multi-)organ dysfunction in patients with strongly elevated CFD plasma level. An important aspect to monitor disease severity is to identify the individual patient risk based on diagnosis, co-morbidity and previous surgical procedures in the light of activation of coagulation and immune responses. An increased C3 and C4 consumption in sepsis, found by inversely lower C3 and C4 levels in patient plasma, is more often associated with unfavorable outcome [31]. Complement activation as an indicator of hospital acquired infection has a strong impact on mortality and hospital stay. Also, the depletion of complement C3 was found to be connected to poor prognosis in severe abdominal sepsis [32].

Our data indicate that the alternative complement pathway interacts with immune processes and coagulation during infection and inflammation. Factor D seems to play a crucial role in this interaction during sepsis. Other authors focused on terminal complement activation and the lectin pathway. High C3, MAC, and MBL serum concentrations are predictive for sepsis-induced disseminated intravascular coagulation [33].

In sepsis, thrombomodulin is a strong predictor of (multi-)organ dysfunction [34]. Hemostasis-related parameters like aPTT, PT and D-dimer are associated with severity of the sepsis [35]. Further, endogenous thrombin can distinguish between beneficial and hazardous hemostatic alterations [36]. Based on the knowledge of dissimilarities in coagulation abnormalities in mice and human, we could not confirm the results of other authors, which found higher D-dimer and TAT complexes associated with disease severity in non-survivors [37].

Based on our findings in the animal experiments regarding dysregulated platelet function, we evaluated the effects of a medication with antiplatelets drugs in our study population. Our data showed reduced CFD plasma level in patients on antiplatelet drugs compared to patients without this medication. So, these could be responsible to improve the septic hypercoagulable state in the patient cohort. These results are in line with the observation in CFD-deficient mice, which shows reduced surface receptor expression on thrombocytes and no relevant platelet drops after sepsis induction over time. Previous studies reported the prevention of organ dysfunction by antiplatelet drugs [38, 39] and low-dose aspirin as a therapeutic option to prevent organ failure [40]. It is hypothesized, that antiplatelet drugs (ASA, clopidogrel) attenuates the expression and release of immune markers (complement factors such as C4b), inflammatory cytokines (including pro- and anti-inflammatory effectors, e.g., TNF-α, IL-1, -6, -10, -13), and induces transcription factor NFκB during inflammation and cellular stress response [41].

### Limitations

The following limitations have to be acknowledged for the present study. (1) The clinical study was designed as retrospective data analysis in a small and selective study group with all its advantages and disadvantages. (2) Patients were recruited from a surgical ICU. Surgical procedures on its own may affect the predictive role of CFD and its impact to in-hospital mortality. More studies are necessary to validate the clinical impact of CFD as potential biomarker and potential discriminator of in-hospital outcome.

## Conclusions

The crosslink between complement and coagulation cascades seems to be permanently present in the course of systemic inflammation. In mice, CFD is linked to pronounced platelet activation, depicted by higher surface expression on thrombocytes in sepsis. In humans, CFD revealed significant impact on in-hospital mortality and predicts disease severity and poor outcome shown by high factor D level in the non-survivor group. The impact of antiplatelet drugs to improve sepsis might be beneficial and affect CFD plasma level and patient outcome.

## Supplementary Information

**Additional file 1.** Complement factor D is linked to platelet activation in human and rodent sepsis.

## Data Availability

The datasets used and/or analysed during the current study are available from the corresponding author on reasonable request.

## Abbreviations

ADP: Adenosine diphosphate
ASA: Acetylsalicylic acid
aPTT: Activated partial thromboplastin time
BW: Body weight
CBA: Cytometric bead array
CD62P: P-selectin
CFD: Complement factor D
CI: Confidence interval
CLP: Cecal ligation and puncture
CRP: C-reactive protein
Ct: Clotting time
C3, -4, -5, -5a: Complement factor 3, -4, -5, -5a
DIC: Disseminated intravascular coagulation
EXTEM: Extrinsic coagulation pathway
FACS: Fluorescence-activated cell sorting
fD^−^^/^^−^: Complement factor D knock out mice
FIBTEM: Fibrinogen function
FITC: Fluorescein-5-isothiocyanate
FXa, -Xia: Factor Xa, -XIa
GPIIb/IIIa: Glycoprotein IIb/IIIa
HR: Hazard ratio
ICU: Intensive care unit
IL-6: Interleukin-6
IL-10: Interleukin-10
INTEM: Intrinsic coagulation pathway
Mcf: Maximal clot firmness
MAC: Membrane attack complex
MBL: Mannose-binding lectin
NFκB: Nuclear factor-kappa B
PE: Phycoerythrin
PMP: Platelet-microparticles
PRP: Platelet-rich-plasma
PT: Prothrombin time
ROTEM: Rotational thromboelastometry
SIRS: Systemic inflammatory response syndrome
SOFA: Sepsis-related Organ Failure Assessment
TAT complex: Thrombin–antithrombin complex
TNFα: Tumor necrosis factor-α
TF: Tissue factor
Wt: Wild-type mice
