# Supplementary material for: Complement factor D is linked to platelet activation in human and rodent sepsis
Source: Intensive Care Med Exp. 2021 Aug 16;9:41. doi: 10.1186/s40635-021-00405-8 (PMC8364893; doi:10.1186/s40635-021-00405-8)
Supplement: Supplementary file 1 — Additional file 1. Complement factor D is linked to platelet activation in human and rodent sepsis. [file 40635_2021_405_MOESM1_ESM.docx]

### Supporting Information

**Complement factor D is linked to platelet activation in human and rodent sepsis.**

Sommerfeld O^1,2,+^, Dahlke K^1,2^, Sossdorf M^1,2^, Claus RA^1,2^, Scherag A^2,3^, Bauer M^1,2^, Bloos F^1,2,+^

^1^Department of Anesthesiology and Intensive Care Medicine, Jena University Hospital, Jena, Germany

^2^Center for Sepsis Control and Care (CSCC), Jena University Hospital, Jena, Germany

^3^Institute of Medical Statistics, Computer and Data Sciences, Jena University Hospital, Jena, Germany

+Correspondence: Frank.Bloos@med.uni-jena.de and Oliver.Sommerfeld@med.uni-jena.de


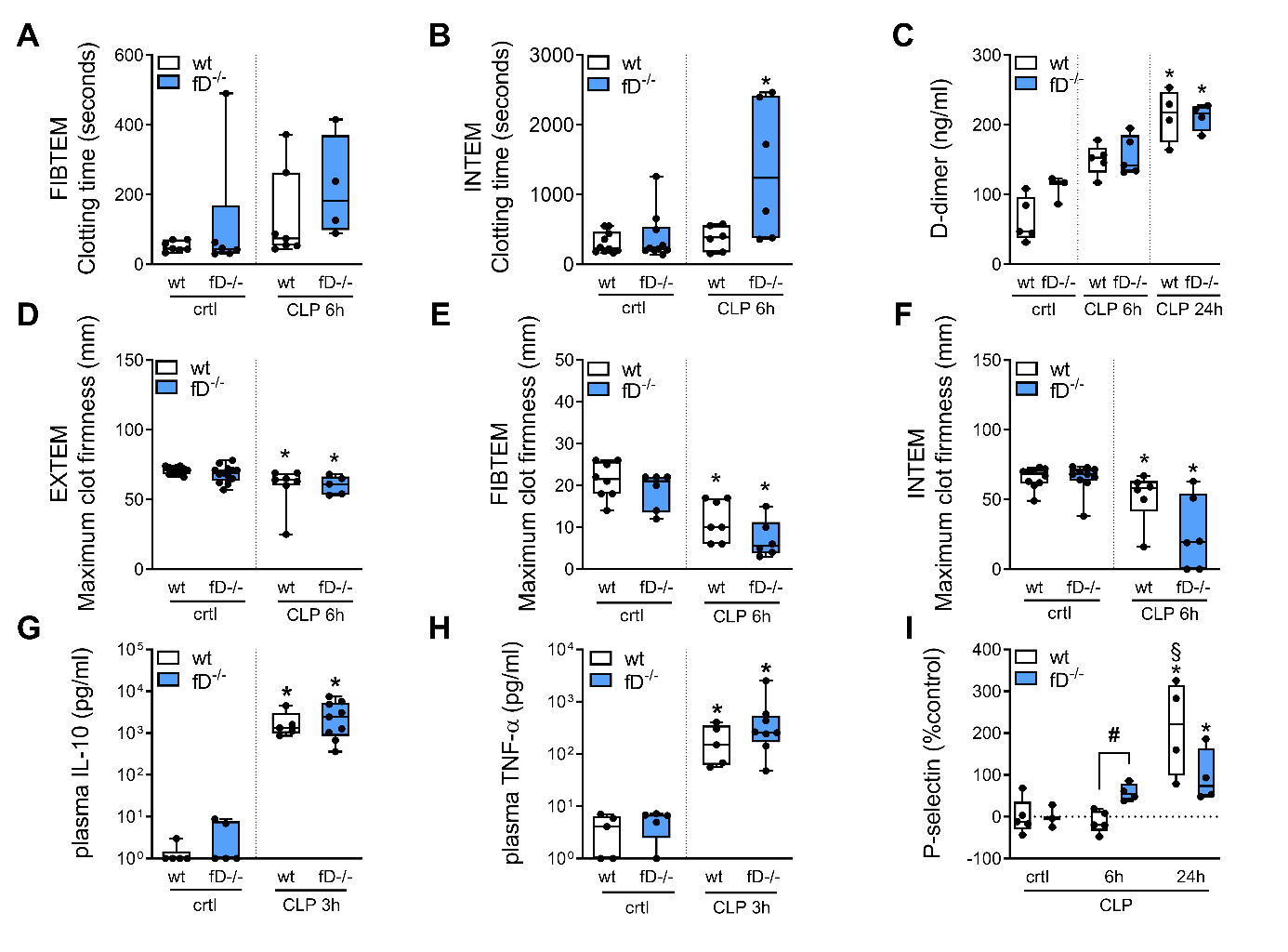


**Figure S1 Thromboelastometry (ROTEM, TEM) and platelet receptor surface expression** (**A**, **B** and **D** to **F**) EXTEM, INTEM and FIBTEM test were performed. (**A** and **B**) Clotting time (ct) in fD^-/-^ mice (fD-/-) was prolonged and (**D** to **F**) maximum clot firmness (mcf) was reduced in both groups after sepsis onset. (**C**) Plasma D-dimer increased over time in both groups after CLP. (**G** and **H**) Plasma cytokine levels of TNF-α and IL-10 increased three hours after CLP. (**I**) Platelet surface expression of P-selectin increased in both groups after CLP.

Statistics: *significant to fD^-/-^ sham control (crtl) or wild-type (wt) sham control (crtl) and §significant to 6 hours wild-type after CLP with *p*<0.05, (**A**, **B** and **D** to **H**) Wilcoxon-Mann-Whitney test or (**C** and **I**) Kruskal-Wallis test (Dunn´s test correction). #Statistical significance (*p*<0.05) between fD^-/-^ and wild-type (wt) by means of (**E**) Wilcoxon-Mann-Whitney test. 3 to 12 mice/group.


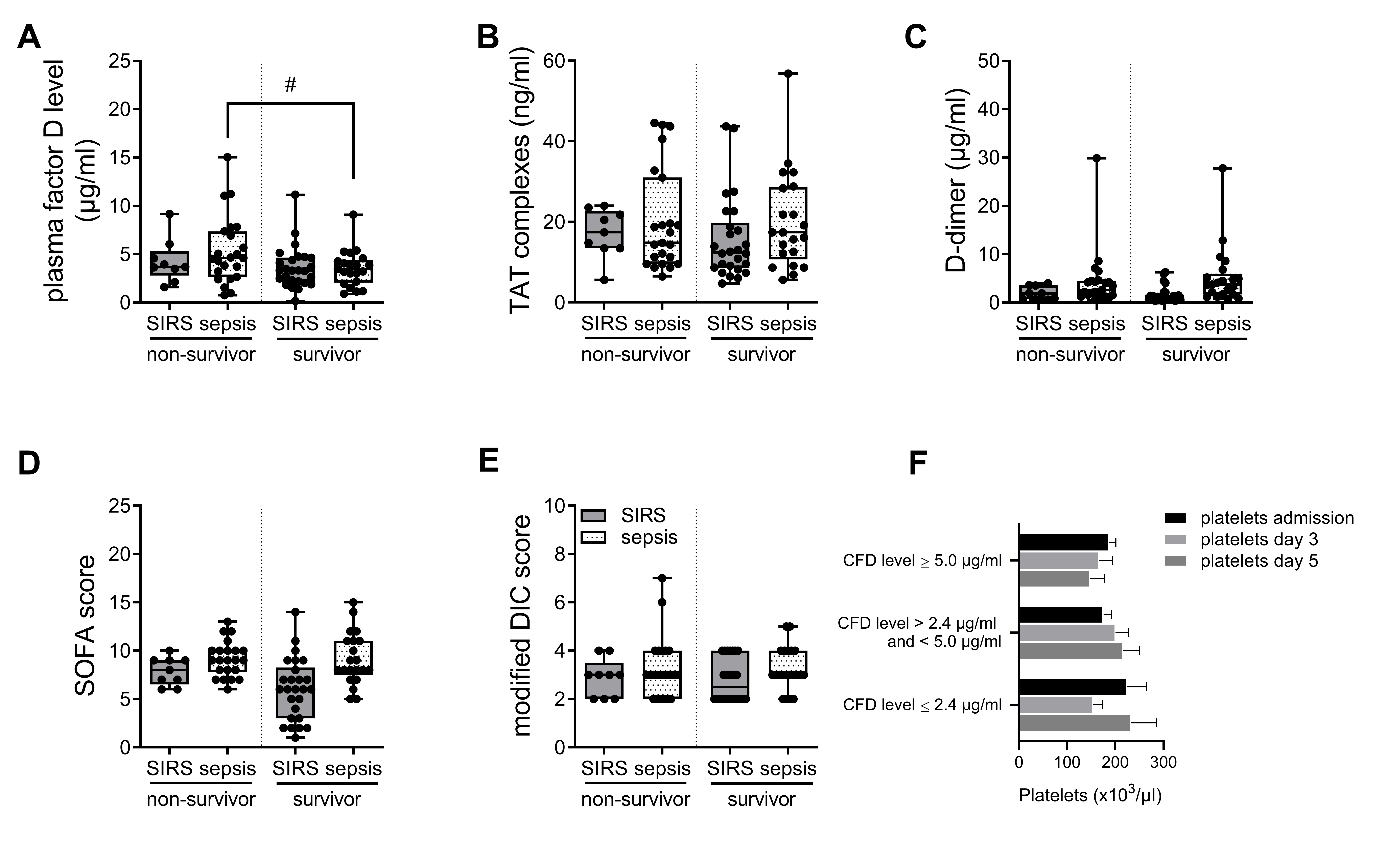


**Figure S2 Coagulation parameters and clinical scores** (**A**) Complement factor D (CFD): Worst outcome was associated with significant higher complement factor D level in septic non-survivors compared to survivor group (SIRS and sepsis). (**B** and **C**) Minor differences were observed for TAT complexes, whereas D-dimer were higher in septic patient (survivor and non-survivor) compared to SIRS survivor group. (**D** and **E**) Higher SOFA score were found in septic patient (survivor and non-survivor) compared to SIRS survivor group. DIC score showed no difference between all groups. (**F**) Platelet count at ICU admission, day 3 and 5 showed minor differences depending to low (≤2.4µg/ml), mid (>2.4 µg/ml and <5.0 µg/ml) or high (≥5.0 µg/ml) CFD plasma concentration.

Statistics: #Statistical significance (*p*<0.05) between non-survivor (sepsis) and survivors (sepsis and SIRS) by means of (**A**) Student’s t-test or (**B** to **E**) Wilcoxon-Mann-Whitney. In total 79 patients.

|  | | | | | | | |
| --- | --- | --- | --- | --- | --- | --- | --- |
|  | | Complement factor D | TAT complexes | D-dimer | Platelets | SOFA score | DIC score (modified) |
| Complement factor D | Pearson correlation | 1 | .101 | -.083 | -.096 | .328^**^ | -.057 |
|  | *p* value |  | .376 | .468 | .399 | .003 | .617 |
|  | N | 79 | 79 | 79 | 79 | 79 | 79 |
| TAT complexes | Pearson correlation | .101 | 1 | .051 | .321^**^ | .082 | -.071 |
|  | *p* value | .376 |  | .658 | .004 | .472 | .536 |
|  | N | 79 | 79 | 79 | 79 | 79 | 79 |
| D-dimer | Pearson correlation | -.083 | .051 | 1 | .141 | .195 | .433^**^ |
|  | *p* value | .468 | .658 |  | .216 | .085 | .000 |
|  | N | 79 | 79 | 79 | 79 | 79 | 79 |
| Platelets | Pearson correlation | -.096 | .321^**^ | .141 | 1 | -.234^*^ | -.232^*^ |
|  | *p* value | .399 | .004 | .216 |  | .038 | .040 |
|  | N | 79 | 79 | 79 | 79 | 79 | 79 |
| SOFA score | Pearson correlation | .328^**^ | .082 | .195 | -.234^*^ | 1 | .330^**^ |
|  | *p* value | .003 | .472 | .085 | .038 |  | .003 |
|  | N | 79 | 79 | 79 | 79 | 79 | 79 |
| DIC score  (modified) | Pearson correlation | -.057 | -.071 | .433^**^ | -.232^*^ | .330^**^ | 1 |
|  | *p* value | .617 | .536 | .000 | .040 | .003 |  |
|  | N | 79 | 79 | 79 | 79 | 79 | 79 |
|  |  |  |  |  |  |  |  |

**Table S1** Results of Pearson correlation analyses of several candidates such as Complement factor D (CFD) level, TAT complexes, D-dimer, platelet count, SOFA score and modified DIC score are plotted in the table. Statistics: **(*p* value ≤0,01) and *(to *p* value ≤0.05) statistical differences for several variables by means of bivariate Pearson correlation coefficients.


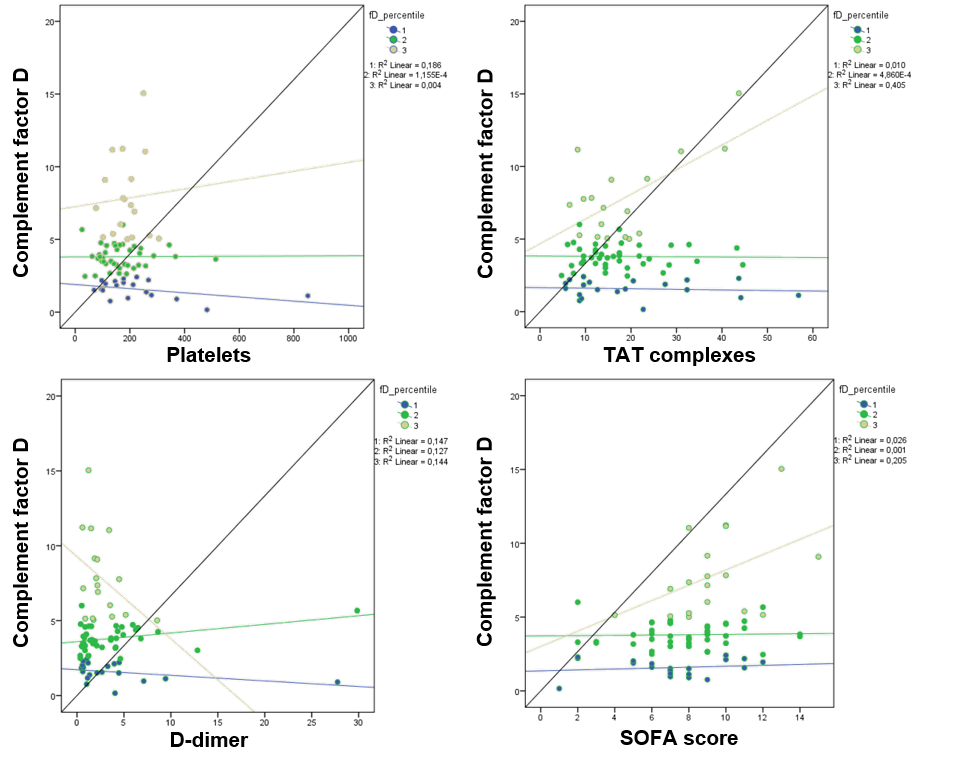


**Figure S3 Correlation analyses** Complement factor D and platelet count showed no correlation. In contrast, complement factor D showed positive correlation to SOFA score. Statistics: All significances are plotted in table S1. Bivariate Pearson correlation coefficients and percentiles (1: 25%, 2: 50% and 3: 75%) are individually marked on the graphs.
